# USP18-Based Negative Feedback Control Is Induced by Type I and Type III Interferons and Specifically Inactivates Interferon α Response

**DOI:** 10.1371/journal.pone.0022200

**Published:** 2011-07-14

**Authors:** Véronique François-Newton, Gabriel Magno de Freitas Almeida, Béatrice Payelle-Brogard, Danièle Monneron, Lydiane Pichard-Garcia, Jacob Piehler, Sandra Pellegrini, Gilles Uzé

**Affiliations:** 1 Institut Pasteur, Cytokine Signaling Unit, Centre National de la Recherche Scientifique, Unité de Recherche Associée 1961, Paris, France; 2 Centre National de la Recherche Scientifique, Unité Mixte de Recherche 5235, University of Montpellier II, Montpellier, France; 3 Institut National de la Santé Et de la Recherche Médicale, Unité 1040, Université Montpellier I, Institut de Recherche en Biothérapie, Hôpital Saint Eloi, Montpellier, France; 4 Division of Biophysics, University of Osnabrück, Osnabrück, Germany; McMaster University, Canada

## Abstract

Type I interferons (IFN) are cytokines that are rapidly secreted upon microbial infections and regulate all aspects of the immune response. In humans 15 type I IFN subtypes exist, of which IFN α2 and IFN β are used in the clinic for treatment of different pathologies. IFN α2 and IFN β are non redundant in their expression and in their potency to exert specific bioactivities. The more recently identified type III IFNs (3 IFN λ or IL-28/IL-29) bind an unrelated cell-type restricted receptor. Downstream of these two receptor complexes is a shared Jak/Stat pathway. Several mechanisms that contribute to the shut down of the IFN-induced signaling have been described at the molecular level. In particular, it has long been known that type I IFN induces the establishment of a desensitized state. In this work we asked how the IFN-induced desensitization integrates into the network built by the multiple type I IFN subtypes and type III IFNs. We show that priming of cells with either type I IFN or type III IFN interferes with the cell's ability to further respond to all IFN α subtypes. Importantly, primed cells are differentially desensitized in that they retain sensitivity to IFN β. We show that USP18 is necessary and sufficient to induce differential desensitization, by impairing the formation of functional binding sites for IFN α2. Our data highlight a new type of differential between IFNs α and IFN β and underline a cross-talk between type I and type III IFN. This cross-talk could shed light on the reported genetic variation in the IFN λ loci, which has been associated with persistence of hepatitis C virus and patient's response to IFN α2 therapy.

## Introduction

Type I and type III (IL-28/29) IFNs form two multigenic families of pathogen-induced cytokines that exhibit common bioactivities through binding to unrelated cell surface receptors [1]. The numerous type I IFN subtypes (α/β/ω) bind to a receptor made of the ubiquitously expressed IFNAR1 and IFNAR2 chains. Conversely, the type III IFNs (λ1, 2, 3) bind to a receptor made of the broadly expressed IL-10R2 and of IFNLR1 (IL-28Rα) whose expression is cell type specific. Therefore, the response to type III IFNs is tissue specific and appears to be mainly restricted to epithelial cells [2].

Downstream of these two receptor complexes is a shared Jak/Stat pathway, involving the Janus kinases Jak1 and Tyk2 that phosphorylate Stat1, Stat2 and Stat3. Activated Stat1/2 associate to IRF9 to yield the ISGF3 complex that induces transcription of IFN-stimulated genes (ISG) [1]. Thus, in humans, the 18 subtypes (13 α, 1 β, 1 ω and 3 λ) of the type I and type III IFN systems induce a same gene subset and exhibit antiviral and antiproliferative activities through two independent cell surface receptors. In addition, the type I IFNs are recognized as mediators linking innate and adaptive immunity *via* their effect on the differentiation and maturation of dendritic cells and T cells, activities not shared with type III IFNs [3].

Among the type I IFNs, the α/ω subtypes on the one hand and the β subtype on the other are not equivalent, as they are differentially produced upon microbial infections and exhibit distinct bioactivities. The biological potency of any given subtype is determined by both receptor binding parameters and receptor density on target cells [4], [5]. Hence, compared to IFN α2, IFN β binds the receptor with higher affinity, forms a longer-lived complex and is more potent at inducing translational control signals, inhibiting cell growth and osteoclastogenesis [6], [7], [8] (Moraga *et al*., submitted). Importantly, IFN α2 is routinely used in the clinic as in chronic HCV infection and several forms of cancer, whereas IFN β is approved for treatment of multiple sclerosis, considered an autoimmune disease [1].

The pleiotropic activities of IFNs must be tightly down regulated in time and space and several mechanisms have been shown to co-exist in order to attenuate IFN-initiated Jak/Stat signaling (reviewed in [9]). In an *in vivo* model, Sarasin *et al* showed that liver cells from mice repeatedly injected with murine IFN α become refractory to further IFN α stimulation [10]. The ISG-encoded isopeptidase USP18/Ubp43 was found to be essential for the establishment of the desensitized state [10], [11]. USP18 can remove the ubiquitin-like ISG15 from target proteins [12] and was found to inhibit IFN-induced Jak/Stat signaling when constitutively expressed in cultured cells [13]. Interestingly, USP18 expression was recently identified as a bad prognostic marker of the success of IFN α therapy in patients with chronic hepatitis C [14], [15].

Here, we have studied how IFN induced desensitization integrates into the network built by the multiple type I and type III IFNs. We found that both type I and type III IFNs can induce a long lasting desensitization state in cells of different lineages, including human primary hepatocytes. Remarkably, the refractory state is targeted to the IFN α and ω subtypes, leaving nearly intact the cells' responsiveness to β and λ IFNs. We show that USP18 is necessary and sufficient to differentially desensitize cells by disturbing the assembly of α IFNs with the receptor complex.

Altogether, our findings emphasize the existence of differential activities within the type I IFN family and underline a novel type I/III IFN cross-talk acting at the receptor level that could have important consequences in the set up of clinical protocols, especially for the treatment of HCV-infected patients who are resistant to conventional pegylated IFN α2 therapy.

## Results

### Type I and type III IFNs induce desensitization to IFN α

**Figure 1:**
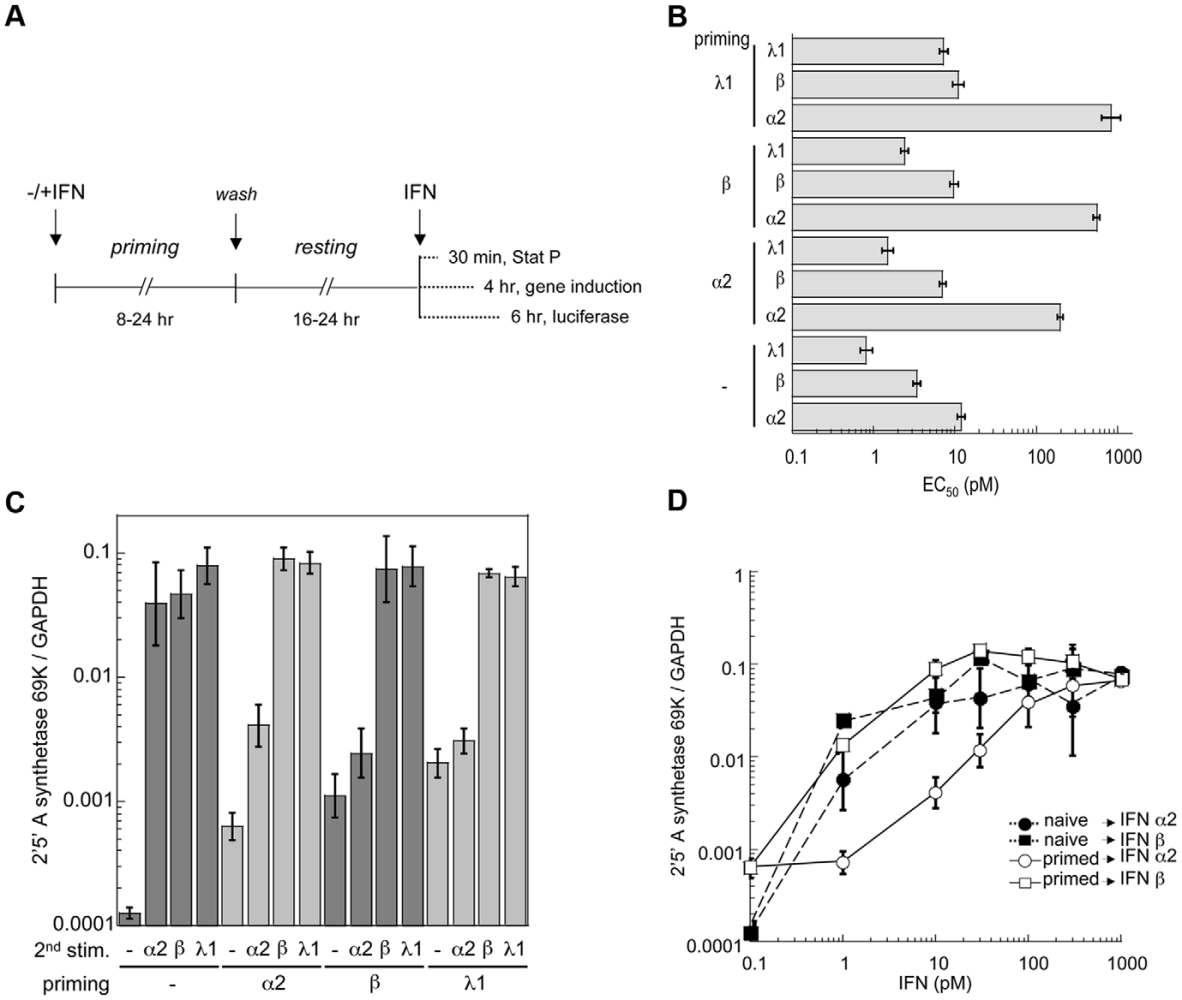
Differential desensitization studies in HLLR1-1.4 cells. (A) Protocol used to measure desensitization. Unless otherwise indicated, cells were primed with IFN α2 or IFN β (500 pM) or IFN λ1 (50 pM). The priming phase varied between 8 and 24 hr and the resting phase between 16 and 24 hr. Cells were then challenged with IFN for different times depending of the read out. (B) Graphic representation of the EC_50_ (pM) as determined by the luciferase activity induced by IFN α2, IFN β or IFN λ1 in naïve or primed cells. EC_50_ were calculated from the non-linear regression fits of the luciferase activity induced by IFN in a concentration range covering 2.4 log. Priming and resting times lasted 24 hr each. Bars represent the 95% confidence limits. (C) Level of *OAS-69K* mRNA induced by IFN α2 (10 pM), IFN β (10 pM) or IFN λ1 (50 pM) in naïve and primed cells as determined by RT-qPCR. Data are expressed as ratios to GAPDH levels. Priming and resting times lasted 24 hr each. Bars represent the 95% confidence limits (Student's t-test). (D) Dose response induction profile of *OAS-69K* mRNA in naïve (closed symbols) and IFN α2 primed cells (open symbols) stimulated for 4 hr with different doses of IFN α2 (circles) or IFN β (squares) as determined by RT-qPCR. Priming and resting times lasted 24 hr each. Data are expressed as ratios to GAPDH levels. Bars represent the 95% confidence limits (Student's t-test).

In a first set of experiments, we established to what extent cells that had responded to IFN α2, IFN β or IFN λ1 could mount a response to a second stimulation. For this, we used HLLR1-1.4 cells, a clone derived from human fibrosarcoma HT-1080 cells stably expressing the IFNLR1 receptor chain and the luciferase reporter gene controlled by an ISGF3-dependent promoter [16]. Thus, HLLR1-1.4 cells are responsive to type I IFNs as well as to type III IFNs (Fig. 1B).

HLLR1-1.4 cells were left untreated (naïve) or stimulated (primed) with IFN α2, IFN β or IFN λ1 for 24 hr, thoroughly washed and kept in fresh medium for another 24 hr (scheme in Fig. 1A). Following this resting period, the levels of Jak/Stat phosphorylation, luciferase activity and *2′–5′ oligo-adenylate-synthetase (OAS 69K)* mRNA in primed cells had nearly returned to basal levels. Naïve and primed HLLR1-1.4 cells were challenged with IFN α2, IFN β or IFN λ1 for 6 hr (Fig. 1A) and luciferase activity was quantified (Fig. 1B). The potency of IFN α2 in luciferase induction (expressed as EC_50_) decreased 14.5–68.9 fold in primed cells as compared to naïve cells, whereas the potency of IFN β decreased only 2.1–3.2 fold (Fig. 1B). A similar trend was observed when naïve and primed cells were monitored by RT-qPCR for induction of *OAS 69K* mRNA in response to 10 pM of each IFN (Fig. 1C). As shown in Fig. 1D, upon desensitization the dose response relationship for IFN α2 had shifted down by a factor of 50-100. Interestingly, the activity of all the α/ω subtypes assayed was decreased in type I and in type III IFN-primed.

**Figure 2:**
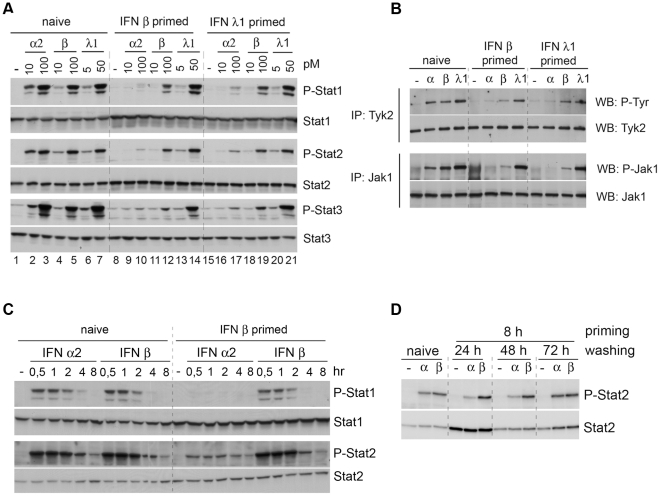
Differential desensitization studies in HLLR1-1.4 cells. (A) Level of phosphorylation of Stat1, Stat2 and Stat3 induced at 30 min by the indicated doses of IFN in naïve cells and in primed cells. Priming was for 8 hr and resting was for 16 hr. Cell lysates (30 µg) were fractionated on a 7% SDS polyacrylamide gel and immunoblotted with the indicated Abs. (B) Level of tyrosine phosphorylation of immunoprecipitated Tyk2 and Jak1 induced at 30 min by IFN α2 (100 pM), IFN β (100 pM) or IFN λ1 (50 pM) in naïve and primed cells. Priming was for 8 hr and resting was for 16 hr. Lysates (2 mg) were immunoprecipitated with Tyk2 Abs (top) or Jak1 Abs (bottom). The top membrane was incubated with phospho-tyrosine 4G10 mAb (P-Tyr) and the bottom membrane with phospho-Jak1 Abs. Protein content was assessed by re-blotting with Tyk2 or Jak1 specific Abs. (C) Kinetics of Stat1, Stat2 and Stat3 phosphorylation in naïve and primed cells. Cells were stimulated with 50 pM of IFN α2 or IFN β, as indicated. Priming was for 8 hr and resting was for 16 hr. Lysates (30 µg) were fractionated on a 7% SDS polyacrylamide gel and immunoblotted with the indicated Abs. (D) Level of phosphorylation of Stat2 induced at 30 min by 100 pM of IFN in naïve cells and in primed cells. Priming was for 8 hr and the resting phase varied from 24 hr to 72 hr. Cell lysates (30 µg) were fractionated on a 7% SDS polyacrylamide gel and immunoblotted with the indicated Abs.

Desensitization was also evident at the level of early signaling events. Fig. 2A shows that cells primed with IFN β or with IFN λ1 were refractory to low doses (10 and 100 pM) of IFN α2 in terms of tyrosine phosphorylation of Stat1, Stat2 and Stat3 (compare lanes 2–3 with lanes 9–10 or 16–17). Conversely, in primed cells Stat1, Stat2 and Stat3 were still phosphorylated upon treatment with IFN β and IFN λ1. Moreover, in primed cells the activation of Jak1 and Tyk2, the earliest effectors of the pathway, was abrogated in the case of IFN α2, but still detectable in the case of IFN β and IFN λ1 (Fig. 2B).

To better characterize the differential desensitization state, we asked if primed cells resumed their response to IFNα2 when stimulated for longer than 30 min. Naïve and primed cells were thus stimulated with IFN α2 and IFN β for up to 8 hr. As shown in Fig. 2C, the desensitization of primed cells to IFN α2 persisted, independently of treatment duration. To define for how long primed cells remained in a refractory state, we extended the interval between priming and restimulation (washing of 24, 48 and 72 hr). As shown in Fig. 2D, primed cells had regained IFN α2 sensitivity after 72 hr in the absence of cytokine. In conclusion, the differential desensitized state of the cell persists even at 8 hr of stimulation, but is reversible as seen when cells are kept in the absence of cytokine for 3 days.

**Figure 3:**
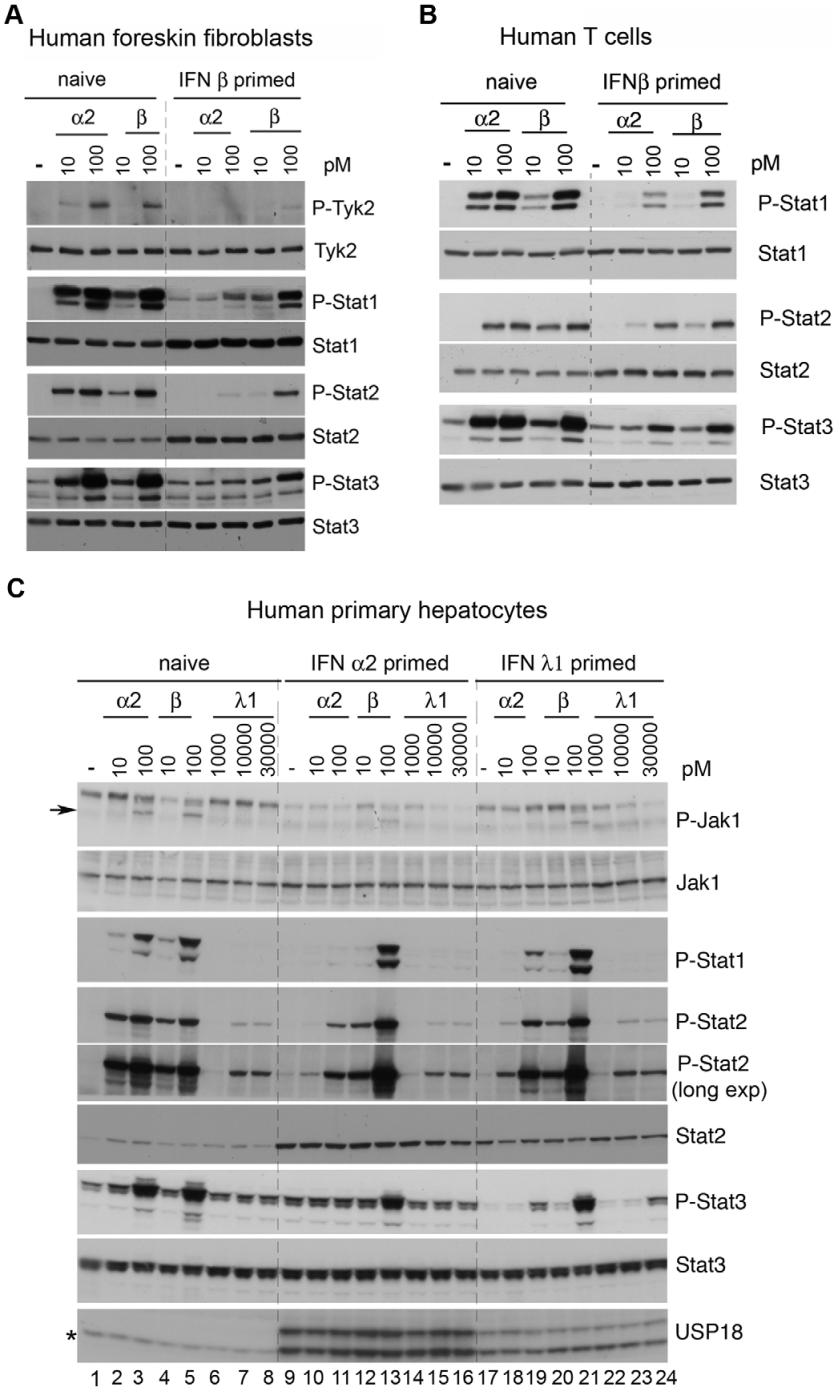
Differential desensitization of human primary cells. (A) Human foreskin fibroblasts and (B) human T cells were either left untreated (naïve) or primed for 8 hr. Cells were washed, maintained in medium without IFN for 16 hr and stimulated for 30 min with 10 and 100 pM of the indicated IFN. Cell lysates (30 µg) were analysed with the indicated Abs. (C) Human primary hepatocytes were left untreated (naïve) or primed with 500 pM of IFN α2 or 30 nM of IFN λ1 for 24 hr. Cells were washed, maintained in medium without IFN for 24 hr and stimulated for 30 min with the indicated IFN doses. Cell lysates (50 µg) were analysed with the indicated Abs to evaluate tyrosine phosphorylation and content of Jak1 and Stats. The arrow points to the band corresponding to phosphorylated Jak1. The level of USP18 (bottom panel) was assessed in a 10% SDS PAGE. Of the two USP18 bands (apparent MW of 38 and 35 kDa), the faster migrating one results from proteolytic processing [46]. This latter comigrates with a non specific cross-reacting band detected in naïve cells and indicated by the asterisk (bottom panel).

Priming with type I IFN induced a similar α2/β differential desensitized state in cell lines, such as bronchial epithelial BEAS-2B and uroepithelial Hs 789.T cells, and in foreskin fibroblasts and T cell blasts (Fig. 3A and 3B). Human primary hepatocytes respond to type I IFNs and more weakly to IFN λ1 (Fig. 3C, compare level of P-Stat2, lanes 1–8). After 24 hr of priming with either IFN α2 or IFN λ1, hepatocytes expressed higher Stat2 protein and detectable levels of USP18, both proteins being encoded by ISG (Fig. 3C, lanes 9–24). Importantly, primed hepatocytes were desensitized to IFN α2 and only marginally to IFN β (Fig. 3C, compare P-Stat2 in lanes 3, 11 and 19) and the extent of desensitization was related to the level of sensitivity to the priming cytokine. Of note, the basal phosphorylation level of Stat3 was reduced in IFN λ1-primed cells with respect to naïve or IFN α2-primed cells (compare P-Stat3 in lanes 1, 9 and 17). Overall, these results demonstrate a previously unrecognized inhibitory cross-talk between the type I and type III IFN systems.

### Desensitized cells are impaired in their ability to bind IFN α2

**Figure 4:**
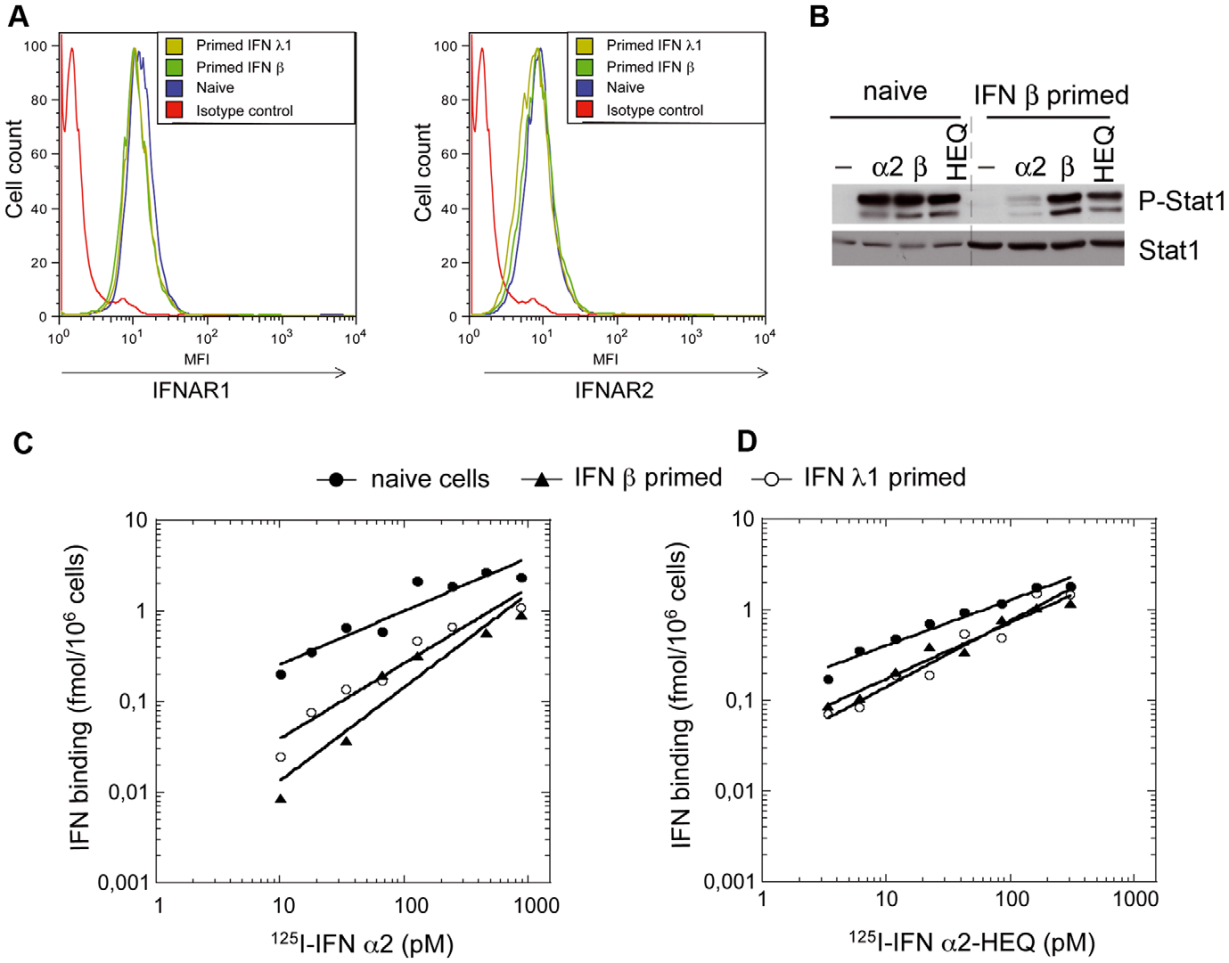
Analysis of the type I IFN receptor in naïve and primed HLLR1-1.4 cells. (A) Surface level of IFNAR1 and IFNAR2 in naïve cells and in IFN β or IFN λ1-primed cells as determined by FACS. Cells were primed for 24 hr, washed and maintained in medium without IFN for 24 hr. Cells were then stained with AA3 mAb (IFNAR1) or CD118 mAb (IFNAR2) followed by biotinylated rat anti-mouse Ab and streptavidin-PE. (B) Level of phosphorylation of Stat1 in naïve and primed cells stimulated for 30 min with 100 pM of IFN α2, IFN β or IFN α2-HEQ. Lysates (30 µg) were immunoblotted with the indicated antibodies. Priming was for 8 hr followed by 16 hr resting in medium without IFN. (C and D) Binding of ^125^I labelled IFN α2 (C) or IFN α2-HEQ (D) at 37°C for 1 hr to naïve (closed circles), IFN β-primed cells (triangles) or IFN λ1-primed cells (open circles). Cells were primed for 8 hr and maintained without IFN for 16 hr.

As shown above, cell desensitization to IFN α2 is manifest at the level of Janus kinase activation and thus may result from reduced surface level of the receptor chains or impaired binding of IFN α2. FACS analysis clearly showed that naïve and primed (*i.e.* desensitized) cells expressed equivalent levels of IFNAR1 and IFNAR2 (Fig. 4A). Therefore, we tested whether the ligand binding property of the receptor differed between naïve and primed cells. For this, we iodinated IFN α2 and, in place of IFN β which cannot be iodinated without loss of bioactivity, we made use of an engineered mutant of IFN α2 (IFN α2-HEQ) whose affinity for IFNAR1 is similar to that of IFN β and which recapitulates IFN β unique biological activities [6]. Accordingly, in primed HLLR1-1.4 cells, IFN α2-HEQ was as potent as IFN β in inducing Stat phosphorylation (Fig. 4B). On this basis, we compared the binding of ^125^I-IFN α2 and ^125^I-IFN α2-HEQ to naïve and to primed HLLR1-1.4 cells. Fig. 4C shows that the binding of ^125^I-IFN α2 was reduced in both IFN β-primed cells and IFN λ1-primed cells with respect to naïve cells. The reduction was most apparent for low ^125^I-IFN α2 concentrations, matching the decrease in specific biological activity (Fig. 1D). In contrast, the binding of ^125^I-IFN α2-HEQ was only marginally reduced in primed cells with respect to naïve cells (Fig. 4D). In conclusion, despite unaltered levels of IFNAR1 and IFNAR2, cells primed with either type I or type III IFNs (*i.e.* desensitized) are unable to assemble a functional IFN α2 receptor complex.

### Expression of USP18 is necessary and sufficient to cause differential desensitization

**Figure 5:**
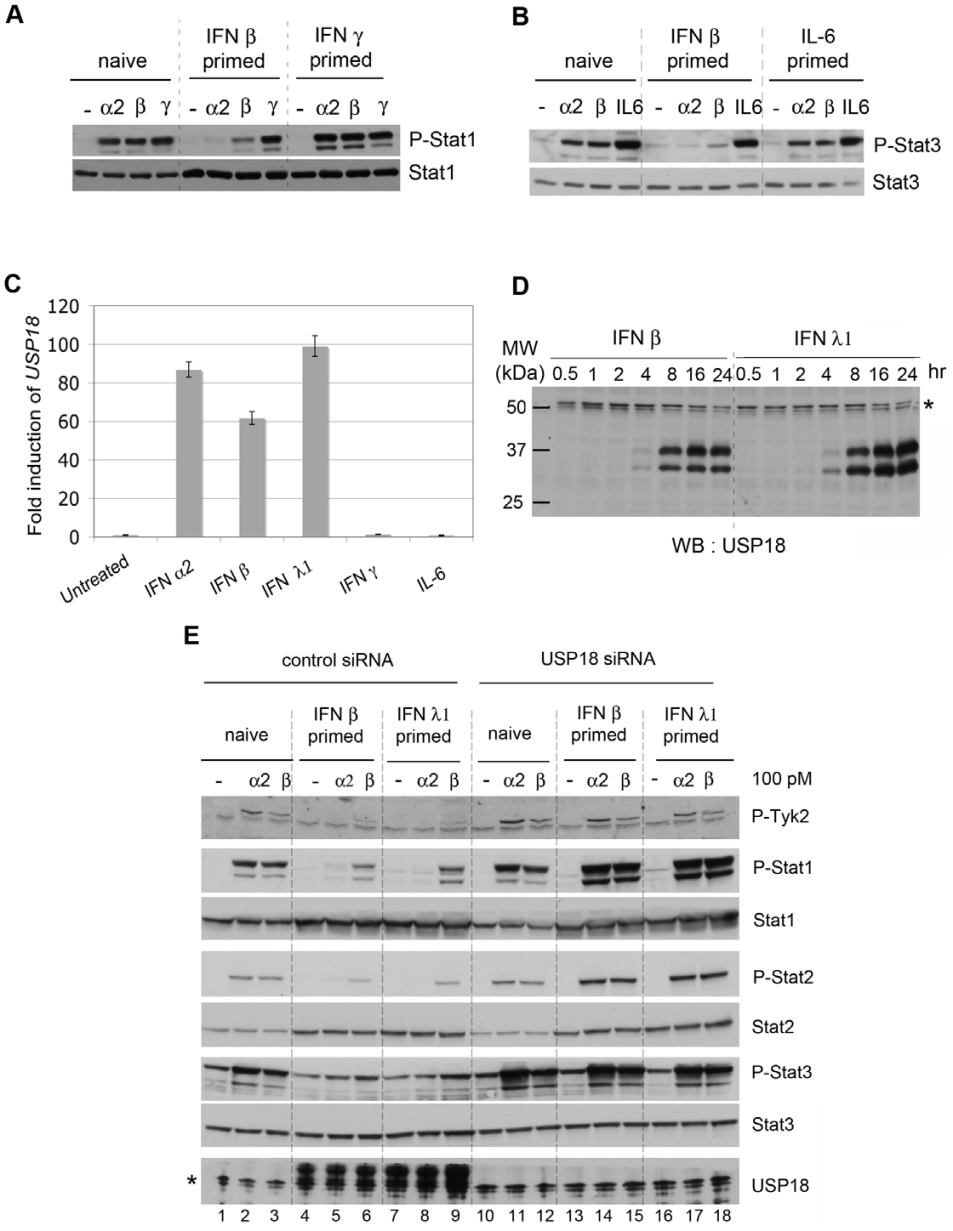
USP18 is necessary for differential desensitization. (A) Stat1 phosphorylation induced in HLLR1-1.4 cells stimulated for 30 min with IFN α2 (100 pM), IFN β (100 pM) or IFN γ (1 ng/ml) in naïve cells and in cells primed with either IFN β (500 pM) or IFN γ (10 ng/ml). Cells were primed for 8 hr and maintained without IFN for 16 hr. (B) Stat3 phosphorylation induced in HLLR1-1.4 stimulated for 30 min with with IFN α2 (100 pM), IFN β (100 pM) or hIL-6 (10 ng/ml) in naïve cells and in cells primed with IFN β (500 pM) or hIL-6 (100 ng/ml). Cells were primed for 8 hr and maintained without IFN for 16 hr. Lysates (30 µg) were immunoblotted with the indicated Abs. (C) Level of *USP18* mRNA in HLLR1-1.4 cells stimulated for 6 hr with IFN α2, IFN β (500 pM), IFN λ1 (50 pM), IFN γ (1 ng/ml) or hIL-6 (100 ng/ml) as determined by qRT-PCR. Each sample was run in triplicate. Transcripts were normalized to the level of 18S transcripts. The ratios between treated and untreated samples in each subset are shown, taking as 1 the ratio in untreated samples. (D) Kinetic profile of USP18 induction in HLLR1-1.4 cells stimulated with 100 pM of IFN β or IFN λ1 for the indicated times. Cell lysates (30 µg) were immunoblotted with the indicated Abs. The asterisk points to a nonspecific band. (E) USP18 is necessary for differential desensitization. HLLR1-1.4 cells were transfected with a control pool of siRNA (Control siRNA) or a pool of four USP18 targeting siRNA (USP18 siRNA). Twenty four hr after transfection, cells were either left untreated (naïve) or primed for 8 hr with the indicated IFN. After 16 hr of resting, cells were stimulated for 30 min with 100 pM of IFN α2 or IFN β. Cell lysates (30 µg) were analysed with the indicated antibodies. The asterisk in the bottom panel points to a band cross-reacting with anti-USP18 Abs (see also USP18 blot in Fig. 3C). Individual USP18 targeting siRNA were also used with similar results (data not shown).

USP18 has been shown to downmodulate type I IFN activity through binding to IFNAR2 [13], and we therefore tested its role in differential desensitization. In HLLR1-1.4 cells *USP18* mRNA was induced by α2, β and λ1 IFNs, but not by other cytokines such as IFN γ or IL-6 (Fig. 5C). Accordingly, cell priming with IFN γ or IL-6 did not induce USP18 nor lead to a desensitized state (Fig. 5A and 5B). USP18 protein accumulated with similar kinetics in cells treated with IFN β or IFN λ1, reaching maximum level between 8 and 16 hr of stimulation (Fig. 5D). As found in primary hepatocytes (Fig. 3C), two USP18-specific bands of comparable intensity were consistently detected.

**Figure 6:**
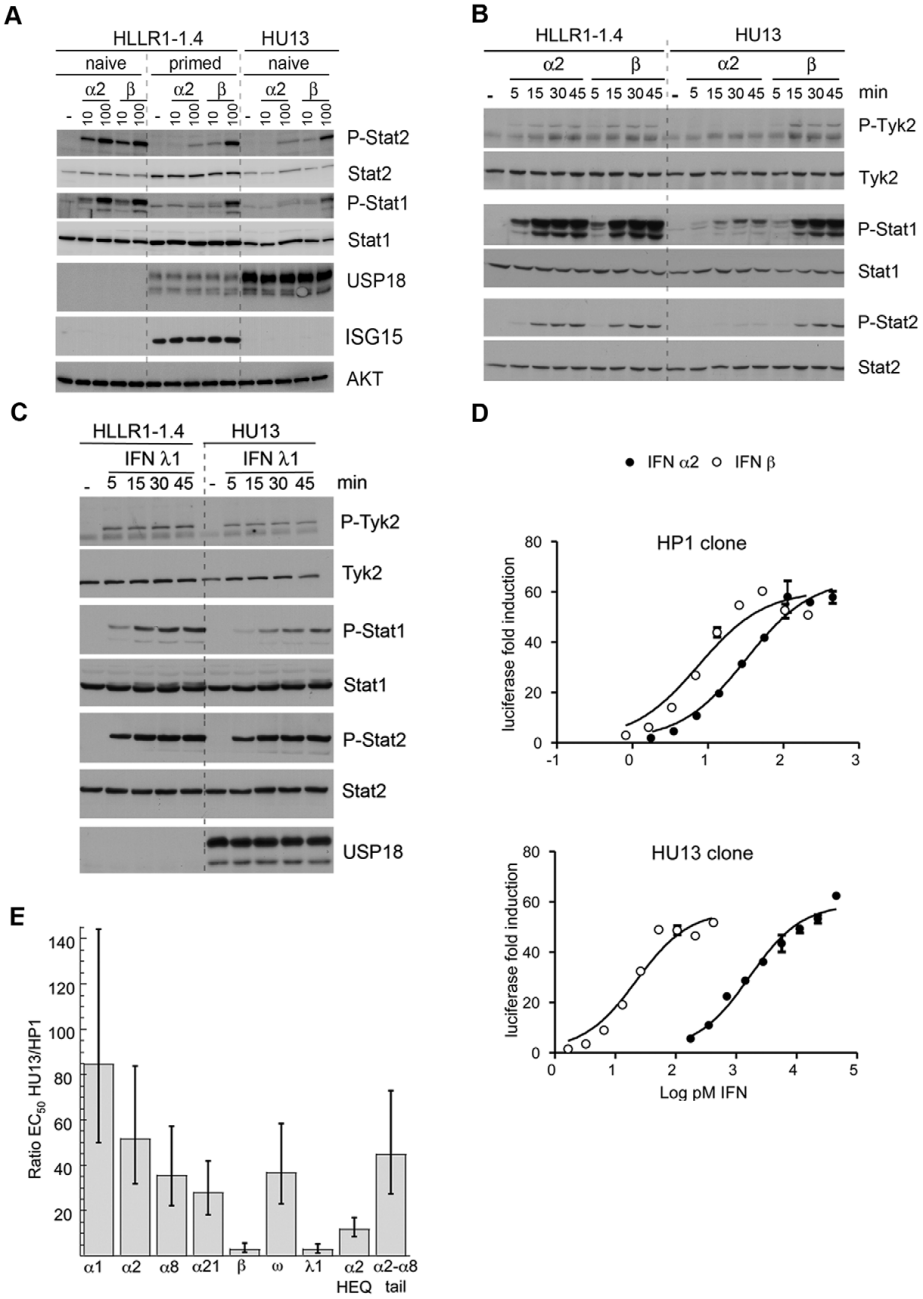
USP18 is sufficient to induce differential desensitization. (A) Level of Stat2 and Stat1 phosphorylation induced by 30 min stimulation with IFN α2 or IFN β in naïve and primed HLLR1-1.4 cells and in clone HU13 stably expressing USP18. Level of USP18 in naïve and primed HLLR1-1.4 cells (endogenous USP18) and in HU13 cells (ectopic USP18). Level of ISG15, a typical ISG, in naïve and primed HLLR1-1.4 and in HU13 cells. Loading was evaluated by measuring AKT. Lysates (30 µg) were immunoblotted with the indicated Abs. (B) Kinetics of Tyk2, Stat1 and Stat2 phosphorylation in the USP18-expressing clone HU13 and in the parental HLLR1-1.4 cells. Cells were stimulated as indicated with 100 pM of IFN α2 or IFN β. Lysates (30 µg) were immunoblotted with the indicated Abs. (C) Kinetics of Tyk2 and Stat1/2 phosphorylation in parental HLLR1-1.4 cells and USP18-expressing HU13 cells. Cells were stimulated as indicated with 30 pM of IFN λ1. (D) Luciferase activity induced by IFN α2 (closed circles) or IFN β (open circles) in HP1 control clone and in HU13 clone constitutively expressing USP18. (E) Ratio of the EC_50_ values determined for luciferase activity on the control clone HP1 and clone HU13. Cells were stimulated with the indicated IFN subtypes for 6 hr. Bars represent support limits of the ratio from 95% confidence intervals of the individual EC_50_.

To study the involvement of USP18, its expression was silenced in HLLR1-1.4 cells (Fig. 5E). Remarkably, cells wherein USP18 was efficiently silenced were not desensitized to IFN α2. Of note, the level of Stat1/2 phosphorylation was higher in USP18-silenced/primed cells (lanes 14, 15 and 17, 18) than in USP18-silenced/naive cells (lanes 11, 12) and most likely this is consequence of the higher content of these Stats in primed cells. To determine if USP18 was sufficient to establish differential desensitization, HLLR1-1.4 cells were stably transfected with a USP18 expression vector or an empty vector. In the 10 positive clones analysed, the level of ectopic USP18 was higher (4 to 50 fold) than endogenous USP18 present in 8 hr-primed cells. Clone HU13 was chosen as it expressed the least USP18 (Fig. 6A). We checked the integrity of the deISGylase activity of USP18 expressed in clone HU13 by comparing the steady state level of ISGylated conjugates in IFN β-treated parental and HU13 cells (data not shown). The response to IFN α2, IFN β and IFN λ1 was measured. Compared to naïve HLLR1-1.4 cells, clone HU13 was severely impaired in its phosphorylation response to IFN α2. Conversely, responses to IFN β and IFN λ1 were preserved or slightly reduced (Fig. 6A–C). By directly comparing luciferase induction in a control clone (HP1) and in HU13 clone, it appeared that constitutive USP18 in HU13 shifted the dose response relationship so that higher concentration of IFN α2 was required to trigger a response of the same magnitude (Fig. 6D). All IFN α subtypes tested and IFN ω were found to be between 25 to 85-fold less potent on HU13 cells than on control HP1 cells, whereas the activities of IFN β and IFN λ1 were marginally reduced (Fig. 6E). The specific activity of IFN α2-HEQ, engineered for higher binding towards IFNAR1 (see above), was reduced only by a factor of 10 on HU13 cells (Fig. 6E), in accordance with the level of activity of this mutant on cells desensitized by IFN priming (Fig. 4B). On the other hand, the specific activity of IFN α2-α8tail, an IFN α2 mutant engineered for higher IFNAR2 binding [7], was decreased by a factor of 45 on HU13 cells as compared to HP1 cells (Fig. 6E). These results indicate that the affinity of a given IFN α subtype towards IFNAR1 determines the degree of USP18-dependent desensitization to that subtype.

**Figure 7:**
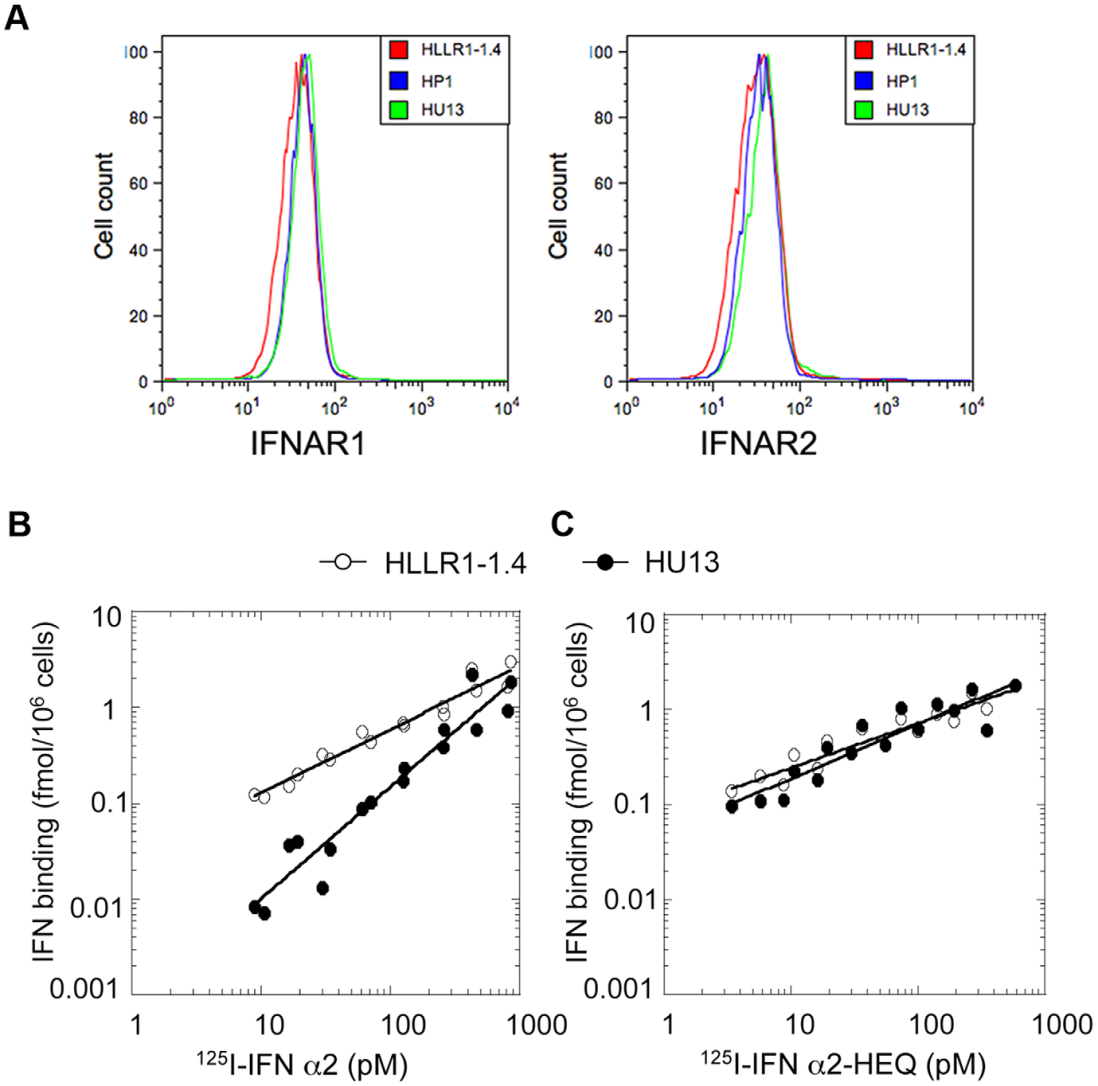
Cells expressing USP18 are defective in IFN α2 binding. (A) Cell surface levels of IFNAR1 (left) and IFNAR2 (right) in parental HLLR1-1.4 cells, USP18-expressing clone HU13 and control clone HP1was determined by FACS. Binding of ^125^I labelled IFN α2 (B), or IFN α2-HEQ (C) at 37°C for 1 hr on HLLR1-1.4 cells (closed circles) and clone HU13 (triangles).

As shown for desensitization caused by IFN priming (Fig. 4A), desensitization caused by expression of USP18 was not dependent on a change of IFNAR1 or IFNAR2 cell surface expression (Fig. 7A). In studies analogous to those performed on IFN-primed cells, we measured binding of radiolabeled ligands on HU13 and naïve HLLR1-1.4 cells. Binding of ^125^I-IFN α2 on HU13 cells was clearly reduced (Fig. 7B) and this to the same extent as for IFN-primed HLLR1-1.4 cells (see Fig. 4C). As expected, the binding of ^125^I-IFN α2-HEQ was similar for the two clones (Fig. 7C). In conclusion, these data demonstrate that the sole expression of USP18 recapitulates the binding alteration observed in IFN-primed (*i.e.* desensitized) cells.

## Discussion

The major findings of our study are hereafter summarized: i) type I IFN and type III IFN desensitize cells to several α IFNs but only marginally to IFN β; ii) cells of different lineages - including primary hepatocytes - undergo differential desensitization; iii) the extent of desensitization is controlled by the level of an ISG, USP18; vi) forced expression of USP18 in naive cells blunts IFN α response at the level of its assembly to the receptor complex.

USP18 is a cysteine protease specialized in the removal of ISG15 from ISGylated proteins. However, the phenotypic alterations caused by USP18 deletion in the mouse have been dissociated from ISG15-dependent mechanisms [17], [18], [19]. One group has proposed that USP18 attenuates IFN α signaling regardless of the isopeptidase activity of the protein by competitively displacing Jak1 from its interaction with IFNAR2 [13]. We have obtained preliminary evidence that, indeed, a catalytically inactive USP18 impairs IFN signaling when highly and stably expressed in naïve cells. In this context, however, desensitization is severe and not differential, as cells become refractory to IFN α and β subtypes. At present, we cannot exclude the possibility that the isopeptidase activity of USP18 could be required in certain physiological contexts, for instance when USP18 is below a given threshold or when the level of ISG15 conjugates is maximal. In fact, while it is remarkable that desensitization can be achieved by constitutive USP18 expression in naïve cells, it is also conceivable that the native protein acquires distinctive biochemical properties - in terms of stability, partners and/or substrates - when it gradually accumulates in IFN-stimulated cells, along with the ISGylation manchinery. On that line, additional work is required to understand the functional link between USP18, ISG15 and the ISGylation machinery in desensitization. Interestingly, several features of the ISG15 system are closely related to the ubiquitin system [20]. Notably, ISG15 is the only ubiquitin-like molecule whose C-terminal residues (LRLRGG) are identical to those of ubiquitin. These similarities suggest functional or regulatory overlap between the two pathways and indeed, *in vitro,* murine USP18 can remove ubiquitin from substrates [21], [22].

From *in vitro* studies, it is known that the assembly of the IFN α2-receptor complex on artificial membranes is conditioned by the IFNAR1 concentration, whereas IFN β recruits IFNAR1 even if present at very low concentration [6]. Indeed, in cells the surface level of IFNAR1 is critical for the intensity of IFN α signaling [5], [23], [24]. Importantly and in accordance with other reports [13], [25], we found that the presence of USP18 has no effect on the global cell surface level of IFNAR2 and IFNAR1. Nonetheless, our study shows that binding of IFN α is impaired in cells expressing USP18, whether IFN-primed or USP18-transfected. Overall, we favor a model whereby the interaction of USP18 with IFNAR2 ([13] and our data) may lead to a re-organization of the architecture of the type I IFN receptor. A change in lateral mobility of the receptor chains, in their localisation in membrane microdomains or their physical preassociation could weaken assembling and signaling of IFN α2. Conversely, owing to its higher affinity for the receptor, IFN β would retain activity on USP18-expressing cells.

It is remarkable that, at least in humans, the 13 α IFNs exhibit non-optimal affinity to the receptor chains and it is precisely this weakness that allows α/β differential bioactivities and desensitization [6]. Thus, in a viral infection, abundant IFN α is likely to be induced from the multiple genes and limits the spread of the virus by exerting potent antiviral action in a timely regulated mode on cells that will then be desensitized. On the other hand, the single IFN β - that is induced alone or, in response to viral infection, co-induced with IFN α [26] - is optimized to bind the receptor chains with high affinity and retains activity on cells desensitized for IFN α. This exclusive property of IFN β may be critical for the stimulation of adaptive immune responses necessary to eradicate the virus.

Type I IFNs and type III IFNs are induced by similar stimuli, exhibit common bioactivities and synergyze in antiviral activity towards several viruses, including HCV [27], [28], [29]. Their functional overlap was somehow expected given the activation, through different receptors, of the same transcriptional factor ISGF3 [30]. This is the first report of an inhibitory effect exerted by IFN λ upon IFN α activities. One particular context where cellular desensitization to IFN α could be relevant is the therapeutical control of chronic HCV infection. The current standard therapy is pegylated-IFN α2 and ribavirin, whose success is influenced by the virus genotype and multiple host factors. Among the strongest predictive factors of treatment outcome is the expression level of ISGs in liver tissue. Indeed, high baseline ISG expression in hepatocytes has been consistently associated with poor response to therapy [14], [15], [31], [32], [33]. Intrahepatic differences in ISG expression may reflect differences in host innate antiviral responses before and/or during the chronic phase. The ISG «signature» is likely to be driven and maintained by local innate cytokines, such as IFNs, and may ultimately result in failure to respond to therapeutic IFN.

Interestingly, USP18 is a component of the gene signature predictive of poor treatment response [15], [32], [33]. Moreover, the knockdown of USP18 in hepatoma cells was shown to potentiate the anti-HCV effect of IFN α [11]. Here, we provide evidence that primary human hepatocytes respond to IFN λ and, when primed with it, they express USP18 and become desensitized to IFN α2. Thus, it is tempting to speculate that this USP18-mediated refractoriness to IFN α could contribute, at least in part, to lower the effectiveness of an IFN α-based therapy. In that event, IFN β or λ would represent alternative therapeutic approaches.

Another strong predictive factor of successful treatment of chronically HCV infected patients (and spontaneous viral clearance) is the IFN λ3 (IL28B) genotype. Paradoxically, the good response IFN λ variant, *ie* predicting higher success rate of IFN α-based therapy, was found to be associated with higher viral load [34], [35], [36], [37]. These consistent observations have spurred intensive studies to try to relate the IFN λ3 (IL28B) genotype with the level of hepatic ISG[33], [38], [39], [40]. To date contradictory conclusions have been reported that do not yet provide a clear picture. Likewise, we are still missing consistent analyses of which of the variants, if any, alters expression level and/or potency of IFN λ.

In view of these and our present data, one can speculate that a patient with the hapless genotype may induce IFN λ inappropriately (e.g. altered level, potency or timing) upon HCV infection. On the one hand, this will lower the viral load without however clearing the virus and, on the other hand, will maintain a high level of ISGs, including USP18. Sustained level of USP18 may contribute, at least in part, to desensitize liver cells to administered IFN α.

## Materials and Methods

### Cells

HLLR1-1.4 cells are described in [16]. HLLR1-1.4 and derived clones were cultured in DMEM with 10% fetal calf serum (FCS), hypoxanthine, thymidine and aminopterin (HAT) and 400 µg/ml G418. To obtain HU and HUS clones, HLLR1-1.4 cells were co-transfected with pSVpuro and pMet7 empty vector or pMet7 encoding USP18 using FuGENE6 (Roche Applied Science). Colonies selected in 0.4 µg/ml puromycin were analysed by immunoblot for USP18 level as compared to primed HLLR1-1.4 cells. Primary hepatocytes were isolated as described [41] from a human liver sample obtained from a 51 y-o female with intrahepatic lithiasis. The French National Ethics Committee has authorized the use of these samples for research. The patient was free of any HCV, HBV and HIV markers at the time of surgery. Hepatocytes were plated at confluence in a 12-well plates at 10^6^ cell/well precoated with collagen in culture medium consisting of Williams' E and Ham's F-12 (Sigma) (1/1 in volume). For the first 24 h, 5% FCS (Gibco) was added to favor cell attachment. The standard medium was then replaced with 1 ml of serum-free medium as described [41]. Cultures were incubated at 37°C and 5% CO2.

### Plasmids and reagents

USP18 cDNA was cloned by PCR using as template the cDNA prepared from HLLR1-1.4 cells stimulated with IFN β-treated for 6 hr and as primers: forward 5′TTTGATATCCTGGGGGTTTTGGAGTGA3′ and reverse 5′TAGACCGGTCTGAAGGTTTTGGGCATTTC 3′. The PCR product was subcloned in pMET7 vector. The catalytic activity of USP18 was assessed by comparing the global protein ISGylation level in 293T cells transiently transfected with ISG15, E1, E2 and E3 enzymes of the ISGylation machinery in the presence or absence of USP18. Rec IFN α2b was a gift of D. Gewert (Wellcome, UK) and IFN β was from Biogen Idec (Boston, MA). Mutants IFN α2-HEQ and IFN α2-α8tail were described in [6], [42]. IFN α1, α8 and α21 were produced as in [43]. IFN ω was from G.R. Adolf (Bender, Wien). All IFNs were purified to homogeneity. Hyper-IL-6, chimeric fusion of human IL-6 and IL-6Rα, was a gift of Merck Serono S.A. Human IFN γ was from PBL, Biochemical Laboratories and IFN λ1 from Peprotech.

### Luciferase reporter assay

To measure luciferase activity, cells were plated in 96-well plate and treated in triplicate for 6 hr with 9 dilutions of IFN in a concentration range covering 2.4 log. Cells were lysed and luciferase activity was quantified in a luminometer (LB960 Berthold). Non-linear regression fits and determination of EC_50_ were done using Prism 5 (GraphPad software).

### Quantitative real-time PCR

Cells were treated with IFN for 4 hr. Total RNA was purified with RNeasy columns (Qiagen). Reverse transcriptions were primed with random primers and performed using Moloney murine leukemia virus reverse transcriptase (Invitrogen). Quantitative real-time PCR (qRT-PCR) was performed using the TaqMan gene expression assay technology (Applied Biosystems) for *USP18* (catalog no. Hs00276441). Each sample was run in triplicate, normalized to the 18S RNA amplification level in the same sample, and calculated relative to the expression of the target gene in unstimulated cells. For measuring *OAS 69K* mRNA, qRT-PCR assays were performed as in [26]. Quantification data are presented as the 95% confidence limits of ratio to the glyceraldehyde 3-phosphate dehydrogenase (GAPDH) level.

### Protein analysis

Cells were processed as in [44]. Polyclonal antibodies (Abs) used were: anti-phospho-Tyr1054/Tyr1055 Tyk2 (Calbiochem); anti-phospho-Tyr689 Stat2; anti-phospho-Tyr701 Stat1, anti-phospho-Tyr705 Stat3, and anti-USP18 (a gift from D.E. Zhang, The Scripps Research Institute, La Jolla, CA), anti-Jak1 (UBI, Lake Placid, NY), anti-Jak1-phospho-YY1022/23 (Biosource, CA) and anti-pan Akt (Cell Signaling Technology, Beverly, MA). Mouse mAbs were anti-Tyk2 T10-2 (Hybridolab, Institut Pasteur, France); anti-phosphotyrosine 4G10 (UBI, Lake Placid, NY) and anti-ISG15 clone 2.1 (a gift from E.C Borden, Cleveland Clinic, Cleveland, Ohio). Signal was revealed with the ECL enhanced chemiluminescence Western blotting reagent (Pierce) or the more sensitive Western Lightning Chemiluminescence Reagent Plus (PerkinElmer). Signals were acquired and quantified with a Kodak Image Station 440 cf. For flow cytometric analyses we used mAbs anti-IFNAR1 AA3 (BiogenIdec, Boston) and anti-IFNAR2 CD118 (P,BL, Piscataway, NJ) or D5 (BiogenIdec, Boston) as in [44]. Samples were analysed with Becton Dickinson FACScan or Canto flow cytometers.

### IFN binding assays

IFN α2 and IFN α2-HEQ (30 µg) were labelled with Iodine^125^ (PerkinElmer, NEZ033A) by using a modified chloramine T method [45]. The labelled IFN preparations were titrated using a luciferase reporter assay relative to IFN α2 and IFN α2-HEQ references of known molar concentrations. The actual incorporations and monomer concentrations were as follows: α2: 75 nM and 54 Bq/fmol; α2-HEQ: 25 nM and 87 Bq/fmol.

For binding assays (Fig. 3C and D), naïve and 8 hr-primed HLLR1-1.4 cells were seeded on 6-well plates (8×10^5^ cells/well) and 16 hr later incubated for 1 hr at 37°C with different concentrations of either ^125^I-IFN α2 or ^125^I-IFN α2-HEQ only or in the presence of a 100 fold excess of unlabeled cold IFN α2-HEQ competitor. Cells were washed three times in DMEM and 5% serum to eliminate unbound IFN, trypsinized, and counted for ^125^I using a γ counter (Berthold). For binding assays on clones (Fig. 6B and C), cells were seeded on 6-well plates (8×10^5^ cells/well) and treated as above.

### USP18 silencing

USP18 ON-TARGETplus SMARTpool and a control siRNA (ON-TARGETplus non-targeting pool) were from Dharmacon. Cells were transfected with 25 nM of siRNA using Lipofectamine RNAi max reagent (Invitrogen), according to manufacturer's instructions. Twenty-four hr later, cells were either left untreated or primed, washed and challenged with IFN α2 or IFN β for 30 min to measure activation of Stats (scheme in Fig. 1A).

The siRNAs constituting the USP18 ON-TARGETplus SMARTpool were also tested individually.
